# Isolation of *Trypanosoma brucei gambiense* from Cured and Relapsed Sleeping Sickness Patients and Adaptation to Laboratory Mice

**DOI:** 10.1371/journal.pntd.0001025

**Published:** 2011-04-19

**Authors:** Patient Pati Pyana, Ipos Ngay Lukusa, Dieudonné Mumba Ngoyi, Nick Van Reet, Marcel Kaiser, Stomy Karhemere Bin Shamamba, Philippe Büscher

**Affiliations:** 1 Institut National de Recherche Biomédicale, Kinshasa Gombe, Democratic Republic of the Congo; 2 Department of Parasitology, Institute of Tropical Medicine, Antwerp, Belgium; 3 Swiss Tropical and Public Health Institute, Basel, Switzerland; 4 University of Basel, Basel, Switzerland; New York University School of Medicine, United States of America

## Abstract

**Background:**

Sleeping sickness due to *Trypanosoma brucei (T.b.) gambiense* is still a major public health problem in some central African countries. Historically, relapse rates around 5% have been observed for treatment with melarsoprol, widely used to treat second stage patients. Later, relapse rates of up to 50% have been recorded in some isolated foci in Angola, Sudan, Uganda and Democratic Republic of the Congo (DRC). Previous investigations are not conclusive on whether decreased sensitivity to melarsoprol is responsible for these high relapse rates. Therefore we aimed to establish a parasite collection isolated from cured as well as from relapsed patients for downstream comparative drug sensitivity profiling. A major constraint for this type of investigation is that *T.b. gambiense* is particularly difficult to isolate and adapt to classical laboratory rodents.

**Methodology/Principal Findings:**

From 360 patients treated in Dipumba hospital, Mbuji-Mayi, D.R. Congo, blood and cerebrospinal fluid (CSF) was collected before treatment. From patients relapsing during the 24 months follow-up, the same specimens were collected. Specimens with confirmed parasite presence were frozen in liquid nitrogen in a mixture of Triladyl, egg yolk and phosphate buffered glucose solution. Isolation was achieved by inoculation of the cryopreserved specimens in *Grammomys surdaster*, *Mastomys natalensis* and SCID mice. Thus, 85 strains were isolated from blood and CSF of 55 patients. Isolation success was highest in *Grammomys surdaster*. Forty strains were adapted to mice. From 12 patients, matched strains were isolated before treatment and after relapse. All strains belong to *T.b. gambiense* type I.

**Conclusions and Significance:**

We established a unique collection of *T.b. gambiense* from cured and relapsed patients, isolated in the same disease focus and within a limited period. This collection is available for genotypic and phenotypic characterisation to investigate the mechanism behind abnormally high treatment failure rates in Mbuji-Mayi, D.R. Congo.

## Author Summary

Human African trypanosomiasis, or sleeping sickness, is still a major public health problem in central Africa. Melarsoprol is widely used for treatment of patients where the parasite has already reached the brain. In some regions in Angola, Sudan, Uganda and Democratic Republic of the Congo, up to half of the patients cannot be cured with melarsoprol. From previous investigations it is not yet clear what causes these high relapse rates. Therefore we aimed to establish a parasite collection isolated from cured as well as relapsed patients for downstream comparative drug sensitivity profiling. From 360 sleeping sickness patients, blood and cerebrospinal fluid (CSF) was collected before treatment and along the prescribed 24 months follow-up. Blood and CSF were inoculated in thicket rats (*Grammomys surdaster*), Natal multimammate mice (*Mastomys natalensis*) and immunodeficient laboratory mice (*Mus musculus*). Thus, we established a unique collection of *Trypanosoma brucei gambiense* type I parasites, isolated in the same disease focus and within a limited period, including 12 matched strains isolated from the same patient before treatment and after relapse. This collection is now available for genotypic and phenotypic characterisation to investigate the mechanism behind abnormally high treatment failure rates in Mbuji-Mayi, Democratic Republic of the Congo.

## Introduction

Within the group of tsetse (*Glossina sp*) transmitted African trypanosomes, *Trypanosoma brucei (T.b.) gambiense* and *T.b. rhodesiense* are the two causative agents of sleeping sickness. This disease, also called human African trypanosomiasis (HAT) constitutes a serious public health threat in Africa, particularly in east and central Africa. In 2006, among 3 million people screened, almost 12,000 HAT patients were diagnosed most of them infected with *T.b. gambiense* [1]. The infection progresses from the first, haemo-lymphatic stage to the second, meningo-encephalitic stage once parasites reach the central nervous system. Without treatment, the disease is almost 100% fatal. No vaccine is expected in the near future [2].

Melarsoprol is still the most widely used drug for treatment of second stage *gambiense* and *rhodesiense* HAT, although for *gambiense* HAT, the combination of eflornithine and nifurtimox is now on the market and will hopefully replace melarsoprol [3]. Historically, an overall relapse rate of 5% has been observed for treatment with melarsoprol [4]. Later, relapse rates of up to 50% after melarsoprol treatment have been noted in some isolated foci in Angola, Sudan, Uganda and Democratic Republic of the Congo (DRC) [5]–[10].

In laboratory models, *in vitro* and *in vivo* resistance of *T.b. brucei* to melarsoprol has been ascribed to reduced uptake of the drug via the P2 nucleoside transporter [11], [12] although the mechanism underlying melarsoprol resistance may be much more complex [13].

Several phenotypic and genotypic studies have been undertaken to explain the alarmingly high relapse rates encountered in the field by reduced sensitivity of *T.b. gambiense* strains to melarsoprol or by mutations in the *TbAT1* gene coding for the P2 transporter [14]–[17]. Though, no conclusive results could be obtained, partially because of bias in parasite strain selection. To confirm or reject the hypothesis of resistance against melarsoprol underlying high relapse rates, it is not sufficient to study parasites from relapsing patients only or from patients with unknown treatment outcome. Rather, comparative *in vitr*o and/or *in vivo* drug sensitivity profiling should be carried out on isolates collected in the same disease focus from both cured and relapsing patients. A major constraint for this type of investigation is the fact that *T.b. gambiense* is particularly difficult to isolate and adapt to rodents and to *in vitro* culture.

In a previous study, we reported on improved isolation of *T.b. gambiense* by inoculation of freshly collected blood and cerebrospinal fluid into the ticket rat *Grammomys (G.) surdaster* [18]. Since these rats are not readily available at places where HAT patients are treated, cryopreservation of patient specimens prior to inoculation into these susceptible rodents is necessary. Maina and co-workers reported on an improved cryopreservation method of *T.b. gambiense* based on a cryomedium for bull semen, Triladyl (MiniTüB, Germany) [19]. By cryopreserving patient blood in Triladyl before inoculation into immunosuppressed *Mastomys (M.) natalensis* and into severe combined immune deficiency (SCID) mice, they isolated with 38% success rate *T.b. gambiense* strains that underwent further genotypic and phenotypic characterisation [14], [20].

Taking advantage of a prospective study on the improvement of follow-up of *gambiense* patients in Mbuji-Mayi (RDC) [6], we decided to isolate *T.b. gambiense* strains from cured patients before treatment and from relapsing patients before treatment and at the time of relapse, in order to establish a unique collection of parasite strains for subsequent comparative drug sensitivity profiling. We here report on the isolation in *G. surdaster*, *M. natalensis* and SCID mice of *T.b. gambiense* strains starting from blood and cerebrospinal fluid (CSF) cryopreserved in Triladyl and on subsequent adaptation of these strains to other breeds of classical laboratory mice (*Mus musculus*).

## Materials and Methods

### Ethics statement

The collection of patients' specimens was approved by the Ethical Committee of the Institute of Tropical Medicine (04441472) and of the Ministry of Health of DRC. Written informed consent was obtained from the patients prior to collection of the specimens. Patients were informed about the objectives and protocol of the study. Experiments on animals were approved by the Veterinary Ethical Committee of the Institute of Tropical Medicine (PAR-022) and adhere to the European Commission Recommendation on guidelines for the accommodation and care of animals used for experimental and other scientific purposes (18 June 2007, 2007/526/EG) and the Belgian National law on the protection of animals under experiment.

### Patients

**Figure 1:**
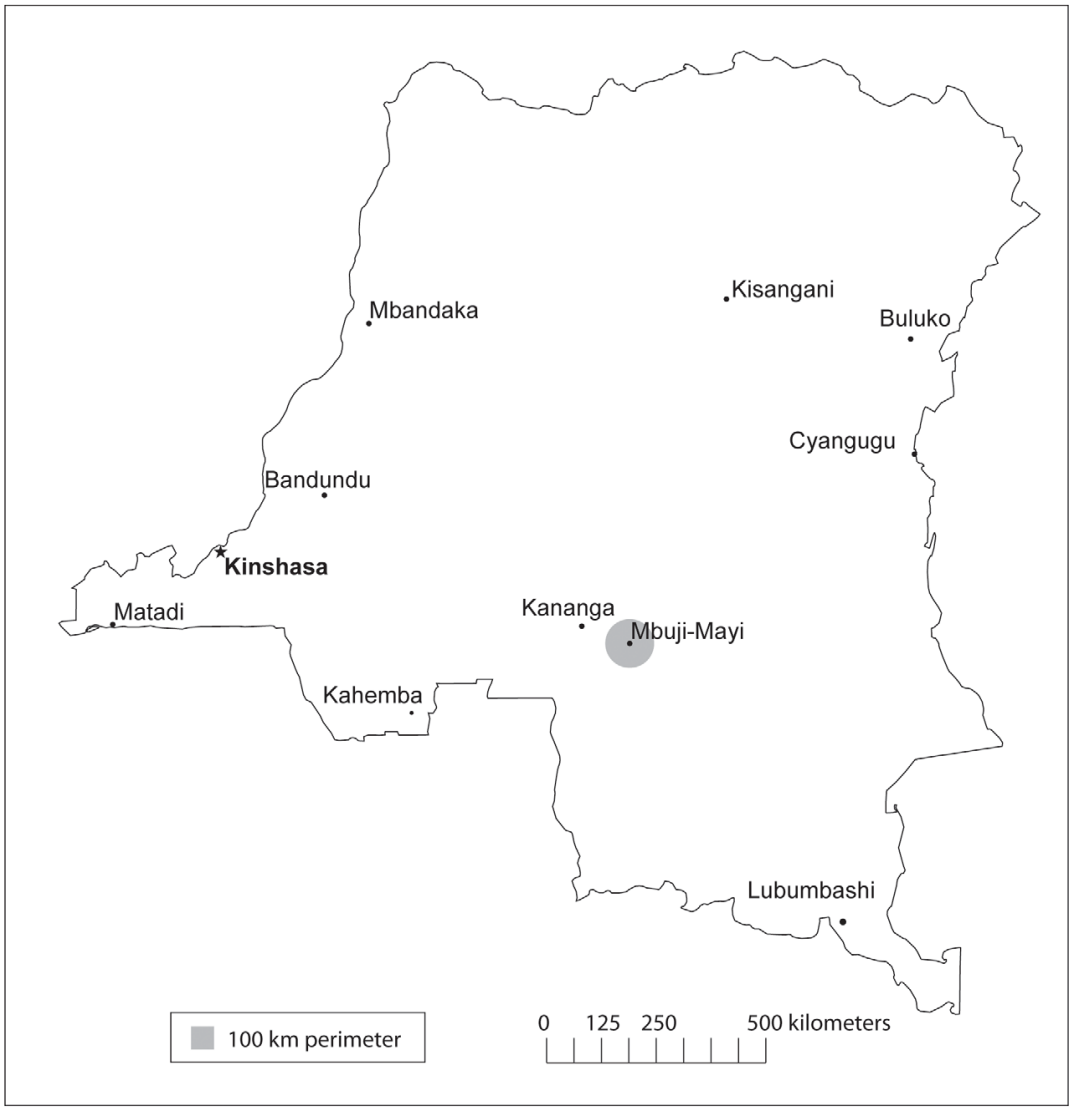
Map of the Democratic Republic of the Congo. The town of Mbuji-Mayi with a 100 km perimeter where the patients in the THARSAT study were recruited (courtesy of Janey Messina, University of North Carolina, Department of Geography).

Patients belong to a cohort included in a follow-up study (THARSAT study) conducted in Dipumba hospital, Mbuji-Mayi, East Kasai Province, DRC between May 2005 and May 2007 (Figure 1). Details on patients, applied treatment regimens, follow-up and treatment outcome definitions have been published elsewhere [6]. Of the 360 patients included, 93 (26%) experienced a confirmed relapse after treatment with a melarsoprol containing regimen, 1 after pentamidine and 1 after eflornithine treatment. Diagnosis at inclusion and at the moment of relapse was confirmed by demonstration of the parasite in lymph node aspirate, venous blood or CSF [6]. This study was approved by the Ethical Committee of the University of Antwerp and of the Ministry of Health of DRC. Written informed consent was obtained from the patients prior to collection of the specimens. Patients were informed about the objectives and protocol of the study. Specimens were collected and cryopreserved from every consenting patient before treatment. Patients were monitored for treatment outcome at 3, 6, 12, 18 and 24 months after treatment. From patients that relapsed, most of them with confirmed recurrence of the parasite, specimens were again cryopreserved.

### Triladyl-egg yolk-phosphate buffered saline glucose (TEP) cryomedium

One volume of aseptically collected egg yolk was mixed with 3 volumes of Triladyl and 3 volumes of phosphate buffered saline glucose (PSG, 7.5 g/l Na_2_HPO_4_.2H_2_O, 0.34 g/l NaH_2_PO_4_.H_2_O, 2.12 g/l NaCl, 10 g/l D-glucose, pH 8). The mixture was divided over 2 ml aliquots and kept frozen at −20°C until use.

### Cryopreservation of venous blood and cerebrospinal fluid

One and a half milliliter of blood on heparin or of CSF was dispensed in microcentrifugation tubes and centrifuged for 5 minutes at 5.200 or 7.244 g, depending on the centrifuge in use. For blood, the plasma was removed and 250 µl of the buffy coat were transferred into 2 ml Sarstedt screw cap micro tubes (Sarstedt, Nümbrecht, Germany) filled with 250 µl of the TEP cryomedium. For CSF, the supernatant was removed leaving about 300 µl of sediment in each tube. The sediment was mixed with 300 µl of TEP cryomedium and 500 µl were transferred into 2 ml Sarstedt screw cap micro tubes. Samples mixed with cryomedium were frozen in the vapour phase of liquid nitrogen for 1 hour where after they were dipped into the liquid phase of the gas until further use. Cryopreserved specimens were shipped to the Institut National de Recherche Biomédicale (INRB) in Kinshasa where isolation in rodents took place.

### Isolation and cryopreservation of parasite strains

Cryopreserved blood and CSF specimens were thawed in a water bath at 37°C and 300 µl were inoculated intraperitoneally (i.p.) into young (1–2 months, both sexes, 20–30 g) *M. natalensis* and adult (9–12 months, both sexes, 30–40 g) *G. surdaster*. Two days before inoculation, the *M. natalensis* were immunosuppressed with 200 mg/kg body weight cyclophosphamide (Endoxan, Baxter, Lessing, Belgium). Immunosuppression with the same dose was repeated after 7 days. *G. surdaster* rats were not immunosuppressed. Parasitemia was monitored three times a week during the first month and further two times a week for at least 2 months by microscopic examination of 5 µl fresh tail blood. When first peak parasitemia was <10^6^/ml, blood from the parasitemic animal was subinoculated into a naive animal which was monitored as described above. When parasitemia reached about 10^6.9^/ml according to the matching method [21], the animal was euthanised with an i.p. injection of sodium pentobarbital at 350 mg/kg body weight (Nembutal, CEVA Santé Animale, Brussels, Belgium). Blood was taken by heart puncture on heparin (Heparin LEO, LEO Pharma, Wilrijk, Belgium) and aliquots of 250 µl were mixed with 250 µl TEP cryomedium and frozen in liquid nitrogen. After one week, one cryovial of each isolate was thawed in a water bath at 37°C and checked in phase contrast microscopy for viability of the cryopreserved trypanosomes. From all isolated strains, copy cryostabilates were deposited in the cryobank of ITM.

### Adaptation of isolates to *Mus (M.) musculus* (NMRI, OF-1, Balb/C and SCID mice)

Adaptation of isolates to laboratory mice took place at INRB in Kinshasa, at the Institute of Tropical Medicine in Antwerp (ITM) and at the Swiss Tropical and Public Health Institute in Basel (STPH). At INRB, adult female NMRI mice were immunosuppressed as described above prior to inoculation. Stabilates were thawed, trypanosome viability was checked under the microscope and from each stabilate 0.5 ml was inoculated i.p. in one mouse. Parasitemia was checked regularly for up to 30 days as described above. Immunosuppression with the same dose was repeated every 5 days. When parasitemia reached 10^7.8^/ml within 12 days post infection, the strain was considered adapted, the mouse was euthanised, blood was taken by heart puncture and 0.2 ml cryostabilates were prepared with TEP cryomedium as described above. At ITM, immune suppressed adult female OF-1 mice were used for adaptation and glycerol 10% was used as cryomedium. At STPH, some isolates were adapted in a similar manner to adult female immunosuppressed Balb/C and to immunodeficient adult female SCID mice. From all adapted strains, copy cryostabilates were deposited in the cryobank of ITM.

### Identification of the subspecies

During adaptation of the strains in *M. musculus*, 200 µl of blood were mixed with an equal volume of AS1 buffer (Qiagen, Hilden, Germany). DNA was extracted with the Maxwell 16 Tissue DNA Purification Kit (Promega, Madison, Wisconsin, USA) and PCR analysed for the *T.b. gambiense* specific TgsGP and for the *T.b. rhodesiense* specific SRA according to the published protocols [22], [23].

## Results

At the moment of inclusion in the study, 41 patients were in first and 319 were in second stage of the disease. From these patients, a total of 436 blood and 588 CSF specimens were cryopreserved before treatment. During the 24 months follow-up, 1 first stage patient and 94 second stage patients experienced a relapse with confirmed presence of trypanosomes. From these relapsed patients, a total of 44 blood and 258 CSF specimens were cryopreserved (table 1). Specimens were cryopreserved immediately after detection of the trypanosomes in order to limit the time between specimen collection and cryopreservation (typically less than 30 minutes). Specimens cryopreserved at Dipumba hospital were shipped within two weeks to INRB where they were transferred from the dry liquid nitrogen shipper into a liquid nitrogen tank.

**Table 1:** Number of patients and relapsing patients and their specimens collected.

			Collected before treatment		Collected at relapse	
Stage	Patients	Relapses	Blood	CSF	Blood	CSF
First	41	1	123	0	4	6
Second	319	94	313	588	40	252
Total	360	95	436	588	44	258

CSF = cerebrospinal fluid.

Isolation of strains into *G. surdaster*, *M. natalensis* and SCID mice started with specimens from the relapsing patients, obtained before treatment and at the time of relapse. Isolation of strains from cured patient specimens collected before treatment could only start with a delay of 24 months when the test-of-cure had taken place and had shown definite cure [6]. Some specimens were inoculated in more than one rodent species and some were inoculated in more than one animal of the same species. Table 2 summarises the isolation results obtained according to the recipient rodent species and the type of specimen inoculated. One hundred fifty one specimens were inoculated in 215 *G. surdaster*, 58 specimens in 109 immunosuppressed *M. natalensis* and 19 specimens in 38 SCID mice of which respectively 81, 17 and 5 became parasitemic. The infection success rate (percentage of animals becoming parasitemic after inoculation) was higher in *G. surdaster* (37.7%) than in *M. natalensis* (15.6%) but not in SCID mice (13.2%). Infection success rates were not different between blood and CSF. Infection success rates were higher in animals inoculated with specimens from cured than from relapsed patients and were respectively 37.5% and 27.1%. The mean time to first observed parasitemia in animals that became parasitemic was 13.8 days post infection in *G. surdaster*, 16.7 days in *M. natalensis* and 19.5 days in SCID with a large variation (minimum 4 and maximum 52 days). Not all strains that grew in the inoculated rodents reached high enough parasitemia levels (≥10^6^/ml) to allow cryostabilisation, even after 2 to 4 subinoculations, but isolation success rate (number of isolates as percentage of inoculated specimens) was much higher in *G. surdaster* (46.4%) than in *M. natalensis* (17.2%) and SCID mice (26.3%).

**Table 2:** Specimens inoculated and isolated in *Grammomys surdaster*, *Mastomys natalensis* and SCID mice.

Rodent	Specimen	Number of specimens	Number of animals inoculated	Number of animals parasitemic	Infection success rate (%)	Days to first parasitemia in parasitemic animals, mean (min-max)	Number of isolated strains (n)	Isolation success rate (%)
*G. surdaster*	Blood	49	76	29	38.2	15.1 (6–52)	25	51.0
	CSF	102	139	52	37.4	13.1 (4–50)	45	44.1
*M. natalensis*	Blood	21	40	8	20.0	17.7 (4–37)	3	14.3
	CSF	37	69	9	13.0	16.0 (6–38)	7	18.9
SCID mouse	CSF	19	38	5	13.2	19.5 (8–31)	5	26.3

Adaptation of the strains isolated in *G. surdaster* and in *M. natalensis* to *M. musculus* occurred at different places and in different breeds, NMRI, Balb/C, OF-1 and SCID. The three first breeds were immunosuppressed before inoculation and during follow-up. In total, 72 animals were inoculated with cryostabilates from which 61 *M. musculus*-adapted populations, representing 45 different strains, could be cryostabilised (Table 3). The adaptation success rate (number of adapted populations as percentage of inoculations) was 81.3% in NMRI, 75.0% in Balb/C, 87.5% in OF-1 and 100.0% in SCID. All mouse adapted strains tested positive in the TgsGP-PCR and negative in SRA-PCR and thus belong to the *T.b. gambiense* type I (data not shown).

**Table 3:** Number of strains inoculated and adapted in different mouse breeds.

Mouse breed	Strains inoculated (n)	Strains adapted (n)	Adaptation success rate (%)
NMRI	32	26	81.3
Balb/C	12	9	75.0
OF-1	16	14	87.5
SCID	12	12	100.0

Table S1 summarises the collection of *T.b. gambiense* strains isolated in Mbuji-Mayi, D.R. Congo, with some of their characteristics. The collection consists of 85 *T.b. gambiense* strains isolated from 1 first stage (patient nr 99) and 54 second stage patients. Fifteen strains were isolated from 13 cured patients and 58 strains from 32 patients that relapsed after 10 days melarsoprol or 14 days melarsoprol-nifurtimox combination treatment. The rest of the strains were isolated from patients with probable relapse (abnormal CSF but not confirmed parasitologically) and from patients with incomplete follow-up. Nineteen strains originate from patients that had already experienced a relapse after melarsoprol treatment before inclusion in the THARSAT study. The majority (58) of the strains could be isolated after a single passage in *G. surdaster*, *M. natalensis* or SCID while for 26 strains two passages and for one strain four passages were necessary. From 12 relapsed patients, matched strains were isolated before treatment and after relapse. Among the 40 strains so far adapted to *M. musculus*, 12 were isolated from cured and 28 from relapsed patients.

## Discussion

The aim of the present study was to establish a collection of *T.b. gambiense* strains isolated from a HAT focus with high treatment failure rates and from relapsed as well as cured patients.

Several methods exist to isolate *T.b. gambiense* from patient specimens. Since it was the intention to isolate bloodstream form trypomastigote populations, methods that result in procyclic populations, like the Kit for In Vitro Inoculation or the feeding of teneral tsetse flies, were not suitable [24], [25]. Inoculation of *in vitro* culture medium containing a feeder layer of *Microtus montanus* embryonic fibroblasts has been described as highly efficient for isolation of bloodstream form trypomastigote populations from CSF [26]. Since technical facilities in Dipumba hospital are rather basic, an in vitro method to isolate bloodstream form trypomastigotes could not be applied in the present study. Instead, we combined two recently described methods for improved isolation of *T.b. gambiense*. The first is based on the observation that *G. surdaster* and immunosuppressed *M. natalensis* are more susceptible to infection with *T.b. gambiense* than *Mus musculus*, the classical laboratory mouse [18]. However, as for SCID mice, it was not feasible to keep these animals close to the patients for the whole study period. The second method allows to cryopreserve patient blood and CSF in liquid nitrogen before inoculation into a susceptible rodent [19]. Thus, it was possible to collect blood and CSF from every patient in the THARSAT study before treatment and to postpone isolation of the parasites until the definite treatment outcome was known, i.e. ≥24 months for cured patients.

Following this protocol, we obtained an isolation success rate of 46.7% in *G. surdaster*, compared to 17.9% in *M. natalensis* and 26.3% in SCID mice. The slightly lower success rate in *G. surdaster* than observed in the former study (62%) can be explained by the cryopreservation of the specimen prior to inoculation [18]. Particularly in specimens with low parasite numbers, the number of live trypanosomes inoculated into the recipient rodent after thawing the cryostabilate may not be sufficient to induce patent parasitaemia. On the other hand, keeping the delay between specimen collection and cryostabiliation short and concentrating the parasites prior to addition of the cryomedium may have contributed to the overall isolation success rate. Noteworthy is that in some cases parasitaemia was only observed more than one month after inoculation. Therefore, a two-month follow-up of the inoculated animals is indicated. The present study confirms the interest to use *G. surdaster* as primary host for *in vivo* isolation of *T.b. gambiense* from HAT patients where after the isolated strains can be further adapted to mice, preferably via SCID mice. To our knowledge, INRB is the only institute with a breeding colony of this thicket rat but because cryopreservation of patient specimens prior to inoculation is compatible with high isolation success, specimens collected in other HAT endemic countries could be sent to Kinshasa for isolation of the parasites in *G. surdaster*.

For downstream genotypic and phenotypic characterisation of isolated strains, it is important to limit the number of passages in rodents as much as possible, although selection bias introduced by cryopreservation and isolation cannot be excluded. Within the current study, most strains underwent only one passage in *G. surdaster* or *M. natalensis* before a primary isolate could be cryostabilised. During further passages in different breeds of *M. musculus*, some strains were lost but for the majority adaptation was straightforward.

The majority of the strains within the collection presented here were isolated from patients that relapsed after treatment with the 10 days melarsoprol treatment recommended by the International Council for Trypanosomiasis Reseach and Control as first line treatment of second stage *T.b. gambiense* patients in 2003 [27]. Within the THARSAT study, patients that relapsed from a previous treatment for HAT were also included [6]. As a result, we were able to isolate strains from patients with multiple relapses after 10 days or 3×3 days melarsoprol (M10 or M3) and 14 days melarsoprol-nifurtimox (MN) treatment, as well as from patients that relapsed after melarsoprol (M10 or M3) treatment and that got cured after subsequent 14 days treatment with eflornithine (E14) or with the melarsoprol-nifurtimox combination. Most importantly, the isolate collection contains 2×12 paired strains isolated from the same patients before treatment and at the time of relapse. We consider this as a major achievement since this will allow precise genotypic and phenotypic comparison of *T.b. gambiense* strains isolated before and after treatment and of strains isolated from cured and relapsed patients. All strains already adapted to *M. musculus* belong to *T.b. gambiense* type I. Based on this finding, coupled to the geographical location of Mbuji-Mayi, we consider the other strains in the collection to be *T.b. gambiense* type I as well. Genetic characterisation using microsatellite markers together with analysis of possible mutations in the *TbAT1* gene, are foreseen during downstream *in vivo* drug sensitivity experiments, so that the observed phenotypic diversity can be directly linked with genetic diversity [28]–[30].

In conclusion, we established a unique collection of *T.b. gambiense* type I populations containing strains from cured and from relapsed patients, isolated in the same HAT focus and within a limited time period. This collection is now available for the research community to conduct detailed genotypic and phenotypic characterisation in order to confirm or reject the hypothesis that resistance to melarsoprol is involved in the abnormally high treatment failure rates observed in Mbuji-Mayi, D.R. Congo.

## Supporting Information

Table S1 List of strains isolated from cured and relapsing patients and their characteristics.
(DOC)
