# Supplementary material for: Isolation of Trypanosoma brucei gambiense from Cured and Relapsed Sleeping Sickness Patients and Adaptation to Laboratory Mice
Source: PLoS Negl Trop Dis. 2011 Apr 19;5(4):e1025. doi: 10.1371/journal.pntd.0001025 (PMC3079580; doi:10.1371/journal.pntd.0001025)
Supplement: Table S1 — List of strains isolated from cured and relapsing patients and their characteristics. (DOC) [file pntd.0001025.s001.doc]

Table S4: List of strains isolated from cured and relapsing patients and their characteristics.

| International code | THARSAT number | Treatment outcome | Time of relapse (month) | Specimen sampling point | Specimen | Passage in rodents before isolation | Adapted to mice | Couple | Prior relapse before inclusion | Treatment received before inclusion | Treatment received at inclusion |
| --- | --- | --- | --- | --- | --- | --- | --- | --- | --- | --- | --- |
| MHOM/CD/INRB/2006/13 | 40 | relapse | 6 | BT | blood | M1G1 | Y | Y | N |  | M10 |
| MHOM/CD/INRB/2006/07 | 40 | relapse | 6 | AT | CSF | M1 | Y | Y | N |  | M10 |
| MHOM/CD/INRB/2008/49 | 104 | relapse | 12 | BT | CSF | G1 | Y | Y | N |  | M10 |
| MHOM/CD/INRB/2008/53A | 104 | relapse | 12 | AT | CSF | G2 | Y | Y | N |  | M10 |
| MHOM/CD/INRB/2008/53B | 104 | relapse | 12 | AT | CSF | G2 | N | Y | N |  | M10 |
| MHOM/CD/STI/2006/02 | 108 | relapse | 6 | AT | CSF | S1 | Y | Y | Y | M10 | MN |
| MHOM/CD/INRB/2007/27 | 108 | relapse | 6 | BT | CSF | G1 | Y | Y | Y | M10 | MN |
| MHOM/CD/INRB/2007/25A | 108 | relapse | 6 | AT | blood | G1 | Y | Y | Y | M10 | MN |
| MHOM/CD/INRB/2007/25B | 108 | relapse | 6 | AT | blood | M1 | Y | Y | Y | M10 | MN |
| MHOM/CD/INRB/2005/02A | 146 | relapse | 3 | BT | CSF | M1 | Y | Y | N |  | M10 |
| MHOM/CD/INRB/2005/02B | 146 | relapse | 3 | BT | CSF | M1G1 | Y | Y | N |  | M10 |
| MHOM/CD/INRB/2006/05 | 146 | relapse | 3 | AT | blood | M1 | Y | Y | N |  | M10 |
| MHOM/CD/INRB/2005/01A | 148 | relapse | 3 | BT | CSF | G1 | Y | Y | N |  | M10 |
| MHOM/CD/INRB/2005/01B | 148 | relapse | 3 | BT | CSF | G2 | Y | Y | N |  | M10 |
| MHOM/CD/INRB/2006/14 | 148 | relapse | 3 | AT | CSF | G1M1 | Y | Y | N |  | M10 |
| MHOM/CD/INRB/2007/29 | 174 | relapse | 3 | AT | CSF | G1 | Y | Y | N |  | M10 |
| MHOM/CD/INRB/2008/59 | 174 | relapse | 3 | BT | CSF | G1 | Y | Y | Y | M3 | M10 |
| MHOM/CD/INRB/2006/16 | 349 | relapse | 3 | BT | CSF | G1 | Y | Y | N |  | M10 |
| MHOM/CD/INRB/2006/19 | 349 | relapse | 3 | AT | blood | G2 | Y | Y | N |  | M10 |
| MHOM/CD/INRB/2007/28 | 57 | relapse | 12 | AT | CSF | G1 | Y | Y | N |  | M10 |
| MHOM/CD/INRB/2008/38 | 57 | relapse | 12 | BT | blood | G1 | N | Y | N |  | M10 |
| MHOM/CD/INRB/2008/50 | 105 | relapse | 3 | BT | blood | G1 | Y | Y | N |  | M10 |
| MHOM/CD/INRB/2008/54 | 105 | relapse | 3 | AT | CSF | G1 | N | Y | N |  | M10 |
| MHOM/CD/INRB/2008/45 | 113 | relapse | 18 | BT | CSF | G1 | N | Y | N |  | M10 |
| MHOM/CD/INRB/2008/60 | 113 | relapse | 18 | AT | CSF | G1 | N | Y | N |  | M10 |
| MHOM/CD/STI/2006/03 | 167 | relapse | 6 | AT | blood | S1 | Y | Y | N |  | M10 |
| MHOM/CD/INRB/2008/43 | 167 | relapse | 6 | AT | CSF | G1 | Y | Y | N |  | M10 |
| MHOM/CD/INRB/2008/47 | 167 | relapse | 6 | BT | blood | G1 | N | Y | N |  | M10 |
| MHOM/CD/INRB/2006/22A | 346 | relapse | 3 | BT | blood | G1 | N | Y | N |  | M10 |
| MHOM/CD/INRB/2007/22B | 346 | relapse | 3 | BT | blood | G2 | N | Y | N |  | M10 |
| MHOM/CD/INRB/2007/24B | 346 | relapse | 3 | AT | CSF | G2 | Y | Y | N |  | M10 |
| MHOM/CD/INRB/2006/44 | 41 | relapse | 18 | BT | blood | G1 | N |  | N |  | M10 |
| MHOM/CD/INRB/2006/18 | 47 | relapse | 6 | BT | CSF | G2 | N |  | Y | M10 | MN |
| MHOM/CD/INRB/2008/52 | 48 | relapse | 6 | BT | blood | G1 | Y |  | N |  | M10 |
| MHOM/CD/INRB/2006/09 | 93 | relapse | 6 | AT | CSF | M1 | Y |  | N |  | M10 |
| MHOM/CD/INRB/2006/11A | 116 | relapse | 3 | AT | CSF | G1 | N |  | N |  | M10 |
| MHOM/CD/INRB/2006/11B | 116 | relapse | 3 | AT | CSF | M1 | Y |  | N |  | M10 |
| MHOM/CD/INRB/2007/11C | 116 | relapse | 3 | AT | CSF | G2 | N |  | N |  | M10 |
| MHOM/CD/INRB/2008/51 | 119 | relapse | 3 | AT | CSF | G1 | N |  | N |  | M10 |
| MHOM/CD/INRB/2007/26A | 147 | relapse | 12 | AT | blood | G1 | Y |  | N |  | M10 |
| MHOM/CD/INRB/2007/26B | 147 | relapse | 12 | AT | blood | G1 | N |  | N |  | M10 |
| MHOM/CD/INRB/2006/06A | 163 | relapse | 3 | AT | blood | M1 | Y |  | Y | M3 | MN |
| MHOM/CD/INRB/2006/06B | 163 | relapse | 3 | AT | blood | G1 | N |  | Y | M3 | MN |
| MHOM/CD/INRB/2008/61 | 169 | relapse | 6 | AT | CSF | G1 | N |  | N |  | M10 |
| MHOM/CD/INRB/2006/17A | 190 | relapse | 6 | BT | CSF | G2 | N |  | Y | M10 | MN |
| MHOM/CD/INRB/2006/17B | 190 | relapse | 6 | BT | CSF | G1 | N |  | Y | M10 | MN |
| MHOM/CD/INRB/2006/12A | 223 | relapse | 3 | AT | CSF | M1G1 | N |  | N |  | M10 |
| MHOM/CD/INRB/2007/12B | 223 | relapse | 3 | AT | CSF | G2 | N |  | N |  | M10 |
| MHOM/CD/INRB/2008/41 | 241 | relapse | 6 | AT | CSF | G1 | N |  | N |  | M10 |
| MHOM/CD/INRB/2006/08 | 242 | relapse | 3 | AT | CSF | M1 | N |  | N |  | M10 |
| MHOM/CD/INRB/2006/10 | 258 | relapse | 3 | AT | CSF | G1 | N |  | N |  | M10 |
| MHOM/CD/INRB/2007/32 | 322 | relapse | 3 | AT | CSF | G2 | N |  | N |  | M10 |
| MHOM/CD/INRB/2006/20 | 336 | relapse | 3 | AT | CSF | G1 | N |  | N |  | M10 |
| MHOM/CD/INRB/2006/21A | 340 | relapse | 3 | AT | CSF | G1 | Y |  | N |  | M10 |
| MHOM/CD/INRB/2006/21B | 340 | relapse | 3 | AT | CSF | G2 | Y |  | N |  | M10 |
| MHOM/CD/INRB/2007/31 | 362 | relapse | 3 | AT | CSF | G1 | N |  | N |  | M10 |
| MHOM/CD/INRB/2007/30 | 371 | relapse | 6 | AT | CSF | G4 | N |  | N |  | M10 |
| MHOM/CD/INRB/2006/15 | 375 | relapse | 3 | BT | blood | G1 | N |  | N |  | M10 |
| MHOM/CD/INRB/2008/56 | 15 | cure |  | BT | CSF | G2 | Y |  | N |  | M10 |
| MHOM/CD/INRB/2008/46 | 19 | cure |  | BT | CSF | G1 | Y |  | N |  | E14 |
| MHOM/CD/INRB/2008/65 | 29 | cure |  | BT | CSF | G2 | N |  | Y | M10 | E14 |
| MHOM/CD/STI/2006/01 | 45 | cure |  | BT | CSF | S1 | Y |  | Y | M3, MN | E14 |
| MHOM/CD/INRB/2008/62 | 85 | cure |  | BT | CSF | G1 | Y |  | Y | M3 | E14 |
| MHOM/CD/INRB/2008/57 | 88 | cure |  | BT | blood | G2 | N |  | N |  | M10 |
| MHOM/CD/INRB/2008/63 | 95 | cure |  | BT | CSF | G1 | Y |  | Y | M10 | MN |
| MHOM/CD/INRB/2008/42***** | 99 | cure |  | BT | blood | G1 | Y |  | N |  | P8 |
| MHOM/CD/INRB/2008/64 | 141 | cure |  | BT | CSF | G1 | Y |  | Y | M10 | MN |
| MHOM/CD/INRB/2005/04 | 145 | cure |  | BT | CSF | G2 | Y |  | N |  | M10 |
| MHOM/CD/INRB/2008/37A | 186 | cure |  | BT | CSF | G1 | Y |  | N |  | M10 |
| MHOM/CD/INRB/2008/37B | 186 | cure |  | BT | CSF | G2 | Y |  | N |  | M10 |
| MHOM/CD/INRB/2006/23A | 348 | cure |  | BT | CSF | G1 | Y |  | N |  | M10 |
| MHOM/CD/INRB/2006/23B | 348 | cure |  | BT | CSF | G2 | Y |  | N |  | M10 |
| MHOM/CD/INRB/2007/34 | 378 | cure |  | BT | CSF | G2 | Y |  | N |  | M10 |
| MHOM/CD/INRB/2008/55 | 27 | probable relapse | 6 | BT | blood | G1 | N |  | N |  | M10 |
| MHOM/CD/INRB/2008/35 | 52 | probable relapse | 12 | BT | CSF | G1 | N |  | N |  | M10 |
| MHOM/CD/STI/2006/02 | 130 | probable relapse | 24 | BT | CSF | S1 | Y |  | Y | MN | E14 |
| MHOM/CD/INRB/2005/03 | 201 | probable relapse | 3 | BT | CSF | G2 | N |  | N |  | M10 |
| MHOM/CD/STI/2006/04 | 16 | withdrawal |  | BT | CSF | S1 | Y |  | Y | M10 | MN |
| MHOM/CD/INRB/2008/40 | 25 | death |  | BT | blood | G1 | N |  | N |  | M10 |
| MHOM/CD/INRB/2007/33 | 347 | death |  | BT | blood | G1 | N |  | N |  | M10 |
| MHOM/CD/INRB/2008/39 | 78 | disappeared |  | BT | CSF | G2 | N |  | Y | M10 | MN |
| MHOM/CD/INRB/2008/58 | 78 | disappeared |  | BT | CSF | G1 | N |  | Y | M10 | MN |
| MHOM/CD/INRB/2008/48 | 117 | withdrawal |  | BT | blood | G1 | N |  | N |  | M10 |
| MHOM/CD/INRB/2008/36A | 181 | disappeared |  | BT | blood | G1 | N |  | N |  | M10 |
| MHOM/CD/INRB/2008/36B | 181 | disappeared |  | BT | blood | G2 | N |  | N |  | M10 |

THARSAT numbers refer to the patient number in the THARSAT study [6]. ***** = strain isolated from a first stage patient. BT = before treatment. AT = after treatment. CSF = cerebrospinal fluid. Y = yes. N = no. SCID = severe combined immuno deficiency. M3 = 3x3 days melarsoprol treatment. M10 = 10 days melarsoprol treatment. MN = 14 days melarsoprol-nifurtimox treatment. P8 = 8 days pentamidine treatment. E14 = 14 days eflornithine treatment. Gx, Mx, Sx: number (x) of passages in *Grammomys surdaster*, *Mastomys natalensis*, SCID mice. Couple = two corresponding strains isolated from the same patient, one before treatment and one at the time of relapse.
